# Hematological and Biochemical Responses During and After a 12-h Track Ultra-Marathon Race

**DOI:** 10.3390/jfmk11020194

**Published:** 2026-05-13

**Authors:** Prokopios Chatzakis, Giorgos Paradisis, Kostas Patas, Stylianos Chatzipanagiotou, Chrysoula Nikolaou, Elias Zacharogiannis

**Affiliations:** 1 School of Physical Education & Sport Science, National and Kapodistrian University of Athens, 17237 Athens, Greece; elzach@phed.uoa.gr; 2 Department of Medical Biopathology, Aeginition Hospital, Medical School, National and Kapodistrian University of Athens, 11528 Athens, Greece; kpatas@eginitio.uoa.gr (K.P.); schatzi@med.uoa.gr (S.C.); xrnikolau@med.uoa.gr (C.N.)

**Keywords:** muscle damage, cardiac biomarkers, inflammation, ultra-marathon

## Abstract

**Objectives**: The purpose of the present study was to examine the acute effects on hematological, inflammatory, cellular, muscular, myocardial, liver, biliary, and humoral immunity biomarkers during and after a 12-h track ultra-marathon event. **Methods**: Twelve healthy male ultra-marathon runners completed the race and all measurements, including venous blood sampling performed before the race (PRE), at 6 h during the race (MID), and immediately after finishing (POST). **Results**: White blood cells, neutrophils, monocytes, basophils, and platelets increased at 6 h (MID) and remained elevated after the finish (POST), while eosinophils and lymphocytes decreased at mid-race and remained suppressed until post-race. The immunoglobulin G and C-reactive protein increased post-race compared to pre- and mid-race values, while lactate dehydrogenase and interleukin-6 increased at mid-race, with no further change until post-race. Creatine kinase, creatine kinase-MB, high-sensitivity cardiac troponin I, aspartate aminotransferase, and alanine aminotransferase significantly increased mid-race and showed a further significant increase post-race. Significant correlations were found between total distance covered and the percentage of PRE-MID difference of interleukin-6 and the percentage of PRE-POST difference of interleukin-6 and lactate dehydrogenase. **Conclusions**: The results of the present study indicate that participation in a 12-h track ultra-marathon is associated with marked exercise-induced alterations in multiple hematological and biochemical biomarkers, with several responses already evident at mid-race (6 h).

## 1. Introduction

An ultra-marathon is typically defined either by a set running distance (exceeding the standard marathon distance) or by a set running time (greater than 6 h, including multi-day events, etc.) [1]. Participation in ultra-endurance events has increased substantially in recent years [2], reflecting growing interest despite the considerable physiological demands imposed by prolonged exercise [3].

Previous research has consistently demonstrated that ultra-marathon participation is associated with alterations in multiple physiological systems. Hematological responses commonly include increases in white blood cells (WBC) and neutrophils (NEU), alongside reductions in hematocrit (HCT) and hemoglobin (HBG) following competition [4,5,6,7]. In parallel, biomarkers associated with skeletal muscle and liver function, such as creatine kinase (CK), aspartate aminotransferase (AST), alanine aminotransferase (ALT), and lactate dehydrogenase (LDH), often show substantial elevations compared to pre-race values [4,5,8,9,10,11,12,13]. Increases in cardiac biomarkers (e.g., troponins, CK-MB, BNP) [4,5,8,10,14,15,16,17,18,19] and inflammatory biomarkers (e.g., CRP and IL-6) [5,8,9,14,15,20,21,22,23] have been widely reported. These responses are generally considered transient and reversible in trained ultra-marathon participants, although their magnitude and time course may vary depending on event characteristics and individual factors [3,11,20]. However, a number of studies suggest that repeated participation in ultra-marathon events may pose long-term health risks for runners [24,25,26].

The 12-h ultra-marathon represents a relatively underexplored model of prolonged endurance exercise. Despite the extensive documentation of physiological responses to ultra-endurance exercise, the temporal evolution of these responses during competition remains insufficiently characterized. Due to the logistical challenges associated with in-race blood sampling in ultra-endurance events, most available evidence is limited to pre- and post-race measurements, thereby limiting insight into the progression of physiological changes during the event itself.

Therefore, the primary aim of the present study was to examine the acute time-course changes in selected hematological and biochemical biomarkers before, during, and immediately after a 12-h track ultra-marathon race. The inclusion of a mid-race measurement provides a valuable opportunity to better characterize the temporal dynamics of these responses under ecologically valid conditions. While the present study is primarily descriptive in nature, it aims to provide a more detailed characterization of the progression of physiological responses during ultra-endurance exercise under real-world conditions. A secondary aim was to explore potential associations between performance (total distance covered) and the magnitude (% change) of biomarker responses across the race.

It was hypothesized that participation in the 12-h ultra-marathon would induce time-dependent alterations in biomarkers, reflecting progressive physiological responses to prolonged exercise (e.g., exercise-induced inflammation, myocardial and muscular damage), and that the magnitude of these responses may be associated with performance-related outcomes.

## 2. Materials and Methods

### 2.1. Participants

Eighteen experienced male ultra-marathon runners (age: 48.78 ± 7.22 years, height: 1.74 ± 0.08 m, body mass: 77.71 ± 10.51 kg) participated in the study. The participants had no recent injuries and were not receiving any medication. They were also regularly trained (≥4 sessions per week) and fully familiarized with ultra-marathon competitions, having completed at least one ultra-marathon race of ≥100 km. All participants were informed about the study and provided written informed consent. The study protocol was approved by the University’s Ethics Committee and conducted in accordance with the Declaration of Helsinki.

### 2.2. Procedures

The competition took place on a 394-m outdoor track and field stadium. Participants were randomly assigned to two groups of nine runners each. The first group started at 7.00 a.m. and finished at 7.00 p.m., while the second group started at 8.00 a.m. and finished at 8.00 p.m. Each runner wore an RFID chip attached to their T-shirt (RaceResult RFID, Pfinztal, Germany) to record lap times, as they crossed over an electronic timing mat, and total distance covered. Athletes ran at self-selected velocity, following their individual pacing strategy. A refreshment station provided water, sports beverages, energy bars, fruits, soft snacks, etc. Food and liquid intake, as well as urine volume, were recorded throughout the race. Metabolic characteristics were evaluated at 3 h, 6 h, 9 h, and 12 h. A member of the research team, riding a light electric vehicle, accompanied each runner and collected the exhaled air for 1 min using a Douglas bag. The gas analysis was performed using a Vacumed gas analyzer (17,620 O_2_ and 17,630 CO_2_ silver edition, Ventura, CA, USA) and a Harvard dry gas meter (Warwickshire, UK), following the standard procedures. Energy expenditure during the 12-h ultra-marathon race was calculated based on the equations of McArdle et al. [27] when VEatps, FECO_2_, and FEO_2_ are known for each workload. A doctor and a nurse were present throughout the race, as well as one hour before the start and one hour after the finish.

Blood samples were collected 1 h before the start (PRE), at 6 h during the race (MID), and immediately after the finish (POST). The mid-race (6-h) time point was selected as it represents the temporal midpoint of the 12-h ultra-marathon event and provided a feasible opportunity to capture in-race physiological responses under field conditions. Venous blood was drawn using standard sterile syringes (BD Emerald 10 mL 21 G) and transferred into collection vials. In addition to the complete blood count, C-reactive protein (CRP) and interleukin-6 (IL-6) were analyzed as inflammatory markers; lactate dehydrogenase (LDH), high-sensitivity cardiac troponin I (cTnI-hs), creatine kinase (CK), and creatine kinase-MB (CK-MB) as cellular, myocardial, and muscular damage markers; aspartate aminotransferase (AST), alanine aminotransferase (ALT), and alkaline phosphatase (ALP) as liver and biliary damage markers; and immunoglobulins A (IgA), G (IgG) and M (IgM) as humoral immunity markers. All samples were stored frozen until analysis.

Complete blood counts were analyzed on an automatic hematology analyzer (CELL-DYN Emerald 22, Abbott, Abbott Park, IL, USA). Liver and biliary damage markers (AST, ALT, ALP), cellular and muscle damage markers (LDH, CK, CK-MB), and CRP were analyzed on an automatic biochemical analyzer (ARCHITECT c 8000, Abbott). The myocardial damage marker (cTnI-hs) was analyzed on an automatic immunological analyzer (ARCHITECT i2000SR, Abbott). IL-6 was analyzed using a Cobas e 411 immunological analyzer (Roche, Basel, Switzerland), and humoral immunity markers (IgA, IgG, IgM) were measured using an automatic BN II nephelometric analyzer (Siemens, Munich, Germany). All analyzers had been previously tested for reliability and validity [28,29,30,31,32].

### 2.3. Statistical Analyses

Results are reported as means ± standard deviation (M ± SD). Data normality was assessed using the Shapiro–Wilk test, while the homogeneity of variances was evaluated using Levene’s test. One-way repeated measures ANOVA was used to determine significant differences in hematological and biochemical parameters across time points [33]. Greenhouse–Geisser corrections were applied when Mauchly’s test of sphericity was violated. When significant effects were detected, Bonferroni post hoc tests were conducted. Cohen’s d and partial eta squared (η^2^) [34] were used as a measure of effect size to assess any significant differences. Effect sizes for differences between in-race measurements using d were determined as trivial (d < 0.20), small (0.20–0.49), moderate (0.50–0.79), or large (>0.80). Concerning the variables RBC, MCV, MCH, BAS, EOS, CRP, and IL-6, the assumption of normality was violated. Therefore, the non-parametric Friedman test was applied. In cases where statistical significance was observed, post hoc pairwise comparisons were conducted using the Wilcoxon signed-rank test with Bonferroni correction [33]. Pearson’s product-moment correlation coefficient (r) was used to find correlations between distance covered during the 12-h ultra-marathon event and the percentage of PRE-MID (%Δ0–6) and PRE-POST (%Δ0–12) difference in all hematological and biochemical parameters measured. Correlations were considered small (r = 0.10–0.29), moderate (r = 0.30–0.49), large (r = 0.50–0.69), or very large (r ≥ 0.70) [35]. Significance for all tests was set at *p* < 0.05. All statistical analyses were performed using SPSS Statistics 25 (IBM SPSS, Inc., Chicago, IL, USA).

## 3. Results

Out of the 18 participants, only 12 completed all the measurements, as 3 of them did not finish the race, and another 3 missed at least one blood test. The mean distance covered was 91.66 ± 17.23 km. Body mass at PRE, MID, and POST was 78.38 ± 3.16 kg, 76.38 ± 3.09 kg, and 75.74 ± 3.16 kg, respectively. Body mass significantly decreased (*p* < 0.001, F = 100.211, partial η^2^ = 0.901) between each successive time point (*p* ≤ 0.006). Total urine output was 0.47 ± 0.36 kg, and total fluid intake was 5.37 ± 2.05 kg, while runners consumed 8235.64 ± 1418.64 kcal through food and drink, and energy expenditure was 8492.08 ± 1429.68 kcal. Significant correlations were found between total distance covered and %Δ0–12_LDH_ (r = 0.593, *p* = 0.042, r^2^ = 0.352, 95% CI = [0.03–0.87]), %Δ0–6_IL-6_ (r = 0.805, *p* = 0.002, r^2^ = 0.648, 95% CI = [0.43–0.94]), and %Δ0–12_IL-6_ (r = 0.778, *p* = 0.003, r^2^ = 0.606, 95% CI = [0.37–0.93]).

Complete blood count results are shown in Table 1. A significant increase at 6 h and post-race finish was found in WBC (F = 44.390, *p* < 0.001, partial η^2^ = 0.801, d = 2.009), NEU (F = 45.106, *p* < 0.001, partial η^2^ = 0.804, d = 2.025), MONO (F = 15.273, *p* < 0.001, partial η^2^ = 0.581, d = 1.18), BAS (χ^2^ = 13.86, *p* = 0.001, Kendall’s W = 0.574), PLT (F = 11.668, *p* < 0.001, partial η^2^ = 0.515, d = 1.030), and PCT (F = 13.194, *p* < 0.001, partial η^2^ = 0.545, d = 1.095), compared to pre-race values. A significant decrease at 6 h and post-race was found in EOS (χ^2^ = 18.24, *p* < 0.001, Kendall’s W = 0.760), and a significant decrease post-race was also found in LYM (F = 7.329, *p* = 0.004, partial η^2^ = 0.400, d = 0.816), compared to pre-race values.

Biochemical responses are shown in Table 2. Significant increases between each successive measurement were observed in AST (F = 11.339, *p* = 0.006, partial η^2^ = 0.508, d = 1.02), ALT (F = 11.012, *p* = 0.006, partial η^2^ = 0.500, d = 1.001), ALP (F = 4.345, *p* = 0.040, partial η^2^ = 0.283, d = 0.63), CK (F = 11.057, *p* = 0.007, partial η^2^ = 0.501, d = 1.003), CK-MB (F = 12.955, *p* = 0.004, partial η^2^ = 0.541, d = 1.09), and cTnI-hs (F = 20.659, *p* < 0.001, partial η^2^ = 0.653, d = 1.37). Significant increases at 6 h and post-race were also found in LDH (F = 10.806, *p* = 0.005, partial η^2^ = 0.496, d = 0.99) and IL-6 (χ^2^ = 19.50, *p* < 0.001, Kendall’s W = 0.813), compared to pre-race values, while CRP (χ^2^ = 20.36, *p* < 0.001, Kendall’s W = 0.85) and IgG (F = 9.820, *p* = 0.004, partial η^2^ = 0.472, d = 0.945) showed significant post-race increases compared to both pre- and mid-race values.

## 4. Discussion

Significant alterations were observed in most hematological and biochemical markers during and after the 12-h track ultra-marathon event. The results of the present study are consistent with our primary hypothesis that significant changes would occur in the biomarkers examined.

More specifically, a significant increase was observed in WBC, NEU, MONO, BAS, and PLT (and consequently PCT), along with a significant decrease in LYM and EOS. Similar hematological responses have been reported in prolonged ultra-endurance events. For example, in a 622-km race, increases in WBC, NEU, MONO, MPV, and MVC and decreases in RBC, HBG, HCT, MCH, and MCHC were observed, with most alterations becoming evident already during mid-race measurements [7]. Likewise, during a 48-h ultra-marathon, WBC, NEU, MONO, PLT, and MCHC increased, whereas MVC and HCT decreased; RBC and HBG remained unchanged immediately after the competition, but decreased during the recovery period [4]. Similar findings have also been reported in other ultra-marathon competitions, where WBC and PLT increased after the race [36,37] and, in some cases, remained elevated during recovery [36], while RBC, HBG, and HCT typically remained unchanged immediately post-race but decreased during recovery [36,37]. Environmental conditions also appear to influence hematological responses, with WBC increasing regardless of conditions, whereas RBC increased and HGB and HCT decreased in hot environments but remained unchanged in cold conditions [38,39].

The increases observed in WBC, NEU, and MONO in the present study are consistent with a typical exercise-induced leukocytosis and likely reflect an acute-phase physiological response to prolonged exercise. This response is likely mediated by mechanisms such as catecholamine-induced leukocyte demargination from the vascular endothelium, cortisol-dependent mobilization from bone marrow stores, and inflammatory signaling associated with skeletal muscle microtrauma during prolonged endurance exercise, as previously described in the literature. The decrease in circulating LYM and EOS concentrations may be explained by exercise-induced redistribution to peripheral tissues combined with cortisol-mediated immunoregulatory effects commonly reported following prolonged endurance exercise. In parallel, the increase in BAS may reflect the activation of inflammatory signaling pathways associated with vascular and immune exercise-induced responses [40,41,42,43].

The increase in PLT observed in the present study may be partly attributed to exercise-induced hemoconcentration, secondary to plasma volume shifts, as well as endothelial activation and inflammatory signaling mechanisms associated with prolonged mechanical loading during ultra-marathon running. In contrast, the absence of significant changes in RBC, HBG, and HCT immediately after the race may be related to the shorter duration of the present event compared with longer ultra-marathon competitions, in which delayed reductions in erythrocyte-related indices are more frequently observed during the rest period.

Significant changes were also observed in IL-6 and CRP, supporting previous evidence that ultra-endurance exercise elicits a pronounced inflammatory response [9,14,16,17]. IL-6 and CRP demonstrated distinct temporal response patterns during the 12-h track ultra-marathon. CRP remained unchanged at mid-race, but increased nearly twelve-fold post-race compared with baseline values, whereas IL-6 increased significantly at mid-race and remained elevated post-race, without any further increase. Similar temporal patterns have been reported in longer ultra-marathon events. For example, during 24-h ultra-marathons, CRP increased progressively from mid-race to post-race measurements [8,19,44], while IL-6 elevations were already evident after the first marathon distance [44]. Likewise, in 100-km ultra-marathon races, CRP typically increases only after the later stages of competition or immediately after the finish [45,46], whereas IL-6 shows an earlier and more progressive increase up to the 75th km [46], findings that are consistent with the present results.

The observed changes may be associated with exercise-induced inflammatory signaling pathways, particularly involving IL-6, which has been suggested to play a role in both metabolic and immunoregulatory regulation during prolonged exercise. In contrast, the delayed increase in CRP is most likely consistent with its role as a downstream hepatic acute-phase protein synthesized primarily in response to IL-6 stimulation. This temporal dissociation between IL-6 and CRP responses may reflect a coordinated cytokine-mediated inflammatory response during prolonged ultra-endurance exercise. Moreover, the elevation in IL-6 concentrations may also partly explain the concurrent leukocytosis observed in the present study, given the known role of IL-6 in modulating immune cell trafficking and activation during prolonged-exercise physiological responses [47,48,49].

LDH, CK, CK-MB, and cTnI-hs increased significantly mid-race, with CK, CK-MB, and cTnI-hs continuing to increase post-race, whereas LDH remained unchanged compared to mid-race values. Similar increases in cardiac troponins (TnI-hs, cTnI, and cTnT) have been reported following various ultra-marathon events [10,15,16,17,19,50,51,52]. Likewise, substantial elevations in CK, CK-MB, and LDH have consistently been observed across ultra-endurance races ranging from 100 km to 768 km [4,8,9,11,12,21,39,45,52,53]. In one of these studies, CK values increased almost one thousand-fold relative to pre-race levels [39], highlighting the magnitude of skeletal muscle stress associated with prolonged ultra-marathon running.

The increases observed in CK and LDH in the present study most likely reflect exercise-induced skeletal muscle disruption resulting from prolonged repetitive mechanical loading and eccentric muscle contractions typical of ultra-marathon running [54]. The track-based format of the race may have further contributed to cumulative musculoskeletal strain due to the repetitive nature of circular running patterns. In parallel, the elevations of CK-MB and cTnI-hs concentrations may indicate transient increases in cardiomyocyte permeability and myocardial strain related to increased cardiac workload during prolonged exercise, as previously reported in ultra-endurance athletes, rather than clinically relevant myocardial injury [55]. Such elevations are generally considered transient and reversible in trained individuals and should be interpreted as expected physiological adaptations rather than indicators of clinical pathology when considered in isolation.

Liver and biliary biomarkers were also affected by the participation in the 12-h track ultra-marathon, particularly AST and ALT, which increased mid-race and further post-race. Similar elevations have been reported after 24-h ultra-marathons [8,9,44], with some studies also documenting post-race increases in ALP, and both total and direct bilirubin [36]. Elevated bilirubin, AST, and ALT levels have been observed in multiple ultra-marathon studies [4,11,12,21,45,53,56,57], supporting the presence of exercise-induced hepatocellular and extrahepatic metabolic responses during prolonged endurance exercise.

However, the increases observed in AST and ALT concentrations following ultra-endurance exercise should be interpreted cautiously, as both enzymes are also present in skeletal muscle tissue and may partly reflect exercise-induced muscle membrane disruption rather than isolated hepatic dysfunction [54]. Prolonged mechanical loading, eccentric muscle contractions, and increased sarcolemmal permeability during ultra-marathon running are known to contribute to the release of intracellular enzymes into the circulation. In addition, sustained metabolic demand and transient reductions in splanchnic blood flow during prolonged exercise may further contribute to mild hepatocellular strain and altered liver enzyme kinetics [58]. The progressive increases observed from mid-race to post-race measurements, therefore, likely reflect the combined effects of skeletal muscle physiological responses to exercise, altered hepatic perfusion, and systemic metabolic load associated with prolonged ultra-endurance performance.

The only humoral immunity marker affected by the 12-h ultra-marathon was IgG. This finding is consistent with previous studies reporting significant post-exercise increases in IgG following 90-km [59] and 100-km [39] ultra-marathon events, while IgA and IgM remained unchanged. The post-exercise increases in immunoglobulins observed after endurance events may reflect an adaptation of the immune system in response to physiological responses induced by exercise [60] and plasma volume shifts associated with prolonged physiological strain [61].

Notably, IgM—the first antibody typically involved in primary immune response following antigen exposure [62]—did not change significantly in the present study, a finding that is consistent with previous reports, suggesting that acute ultra-endurance exercise does not consistently alter circulating IgM concentrations. In contrast, increases in IgG concentrations during prolonged exercise may be partly influenced by cytokine-mediated immunoregulatory responses involving T-helper-2-related cytokines such as IL-4, IL-6, and IL-10, as well as the cortisol-dependent modulation of B-cell activity [47,59]. In addition, exercise-induced hemoconcentration resulting from plasma volume reductions during prolonged running may further contribute to the observed elevation in circulating immunoglobulin concentrations [61]. Collectively, these mechanisms may explain the humoral immune response pattern observed in the present study.

The present findings may have practical implications for the monitoring of ultra-endurance athletes during prolonged events. Importantly, the inclusion of a mid-race measurement provides an improved temporal resolution of biomarker responses during competition, allowing for a more detailed characterization of the onset and progression of these physiological responses, rather than the identification of novel underlying mechanisms. The early elevation of key hematological and biochemical biomarkers at mid-race suggests that measurable physiological responses develop well before race completion. This may be relevant for in-race monitoring strategies aimed at assessing physiological load and fatigue progression. However, it should be noted that clear clinical thresholds for exercise-induced elevations in several of the measured biomarkers are not well established in ultra-marathon contexts. Therefore, interpretation should focus on relative changes and temporal patterns rather than absolute diagnostic cut-offs. These findings may also inform post-race recovery considerations, highlighting that physiological stress responses are already pronounced during the early and mid-stages of ultra-endurance events. Nevertheless, inter-individual variability and the transient nature of these responses must be taken into account when translating these findings into practice.

The findings of the present study should be interpreted in light of certain limitations. The relatively small sample size and the inclusion of only participants who completed all measurements may introduce survivorship bias, limiting generalizability. In addition, the field-based design did not allow for the standardization of pacing strategies, nutritional intake, hydration status, or environmental exposure. However, this design aimed at preserving ecological validity and replicating real competition conditions. The track-based format of the present study differs from typical trail or road ultra-marathon events, as it is characterized by reduced terrain variability and repetitive biomechanical loading patterns. These features may influence the magnitude and distribution of physiological responses compared to more heterogeneous ultra-endurance race environments. While this approach enhances the applicability of the findings to real-world ultra-endurance events, it may also introduce variability in biomarker responses as fluid intake and exercise-induced changes in hydration status may have influenced circulating biomarker concentrations through plasma volume shifts, potentially contributing to hemoconcentration or hemodilution effects. Furthermore, the relatively small sample size limited the use of more complex statistical approaches capable of capturing individual response trajectories, which should be addressed in future research with larger cohorts. Additionally, the descriptive nature of the analytical approach limits the ability to derive mechanistic or causal interpretations from the observed responses. Finally, the absence of post-race recovery measurements limits the ability to fully characterize the time course of biomarker normalization.

## 5. Conclusions

In conclusion, participation in a 12-h track ultra-marathon is associated with alterations in multiple hematological and biochemical markers, reflecting expected physiological responses to prolonged endurance exercise. Importantly, several biomarkers were already elevated at mid-race (after 6 h of running), indicating that these responses develop early during competition.

The inclusion of an in-race measurement provides additional insight into the onset of these physiological responses, which cannot be captured through pre–post designs alone. The observed associations between performance and biomarker responses should be considered exploratory. Overall, the findings should be interpreted in light of the observational and field-based design of the study, which prioritized ecological validity over strict experimental control of key variables.

Taken together, the present findings provide insight into the temporal dynamics of hematological and biochemical responses during ultra-endurance exercise under real-competition conditions, while also highlighting methodological constraints inherent to field-based research designs. These findings should be considered primarily descriptive and exploratory, providing a foundation for future hypothesis-driven and mechanistic research. Ultra-marathon participation elicits measurable physiological responses from the early stages of competition and highlights the importance of appropriate and well-structured recovery strategies following ultra-endurance events. Future research incorporating larger cohorts and extended recovery monitoring is warranted to further clarify the magnitude, variability, and practical implications of these responses in relation to athlete health and performance, as well as incorporating mixed-effects modelling or individual response analyses to better characterize heterogeneity in physiological responses.

## Figures and Tables

**Table 1:** Complete blood count PRE, MID, and POST the 12-h ultra-marathon race.

Variable	PRE	MID	POST	*p* Value	%Δ0–6	%Δ0–12
RBC (10^6^/μL)	5.37 ± 0.58	5.62 ± 0.81	5.62 ± 0.72	0.097	4.7 ± 10.5	4.8 ± 8.9
HBG (g/dL)	14.19 ± 0.96	14.32 ± 1.11	14.45 ± 1.33	0.330	0.9 ± 3.2	1.8 ± 5.3
HCT (%)	43.51 ± 2.67	43.93 ± 2.93	44.38 ± 3.14	0.206	1.0 ± 3.5	2.0 ± 4.7
MCV (fL)	80.10 ± 12.18	79.63 ± 12.10	80.09 ± 11.42	0.079	−0.6 ± 0. 8	0.2 ± 1.4
MCH (pg)	26.09 ± 4.25	26.00 ± 4.28	26.16 ± 4.36	0.148	−0.4 ± 1.5	0.2 ± 1.7
MCHC (g/dL)	32.68 ± 0.66	32.58 ± 0.62	32.54 ± 1.03	0.784	−0.3 ± 1.7	−0.4 ± 2.7
WBC (10^3^/μL)	6.65 ± 1.28	14.61 ± 4.08 *	16.40 ± 4.46 *	<0.001	122.2 ± 57.3	151.3 ± 66.5
NEU (10^9^/L)	3.23 ± 0.90	11.56 ± 4.09 *	13.38 ± 4.53 *	<0.001	265.7 ± 119.2	330.4 ± 144.2
LYM (10^9^/L)	2.68 ± 0.46	2.07 ± 0.66	1.96 ± 0.65 *	0.004	−20.5 ± 29.1	−25.5 ± 24.3
MONO	0.48 ± 0.12	0.74 ± 0.24 *	0.83 ± 0.19 *	<0.001	62.8 ± 55.7	85.0 ± 63.7
EOS	0.23 ± 0.08	0.09 ± 0.03 *	0.08 ± 0.04 *	<0.001	−51.4 ± 27.9	−55.6 ± 31.3
BAS	0.06 ± 0.05	0.14 ± 0.05 *	0.16 ± 0.05 *	0.001	133.3 ± 211.7	166.7 ± 237.3
PLT (10^3^ μL)	243.3 ± 55.4	290.2 ± 84.1 *	290.5 ± 68.5 *	<0.001	18.1 ± 13.5	20.4 ± 16.1
PCT (%)	0.22 ± 0.04	0.27 ± 0.06 *	0.26 ± 0.04 *	<0.001	21.0± 15.1	20.4 ± 15.9
MPV (fL)	9.31 ± 1.27	9.45 ± 1.24	9.33 ± 1.27	0.614	1.7 ± 6.0	0.4 ± 6.1

* Significant difference from PRE.

**Table 2:** Biochemical markers PRE, MID, and POST the 12-h ultra-marathon race.

Variable	PRE	MID	POST	*p* Value	%Δ0–6	%Δ0–12
AST (U/L)	24.7 ± 4.1	37.2 ± 8.6 *	101.5 ± 78.5 *#	0.006	50.2 ± 19.9	288.7 ± 248.8
ALT (U/L)	22.5 ± 7.1	25.8 ± 7.5 *	36.8 ± 16.6 *#	0.006	15.9 ± 11.4	66.1 ± 60.3
ALP (U/L)	51.3 ± 15.3	54.5 ± 13.3	56.1 ± 12.9	0.040	8.1 ± 11.9	11.6 ± 10.7
CK (U/L)	176 ± 94	552 ± 295 *	3402 ± 3336 *#	0.007	227.2 ± 104.0	1726 ± 1169
CK-MB (U/L)	12.50 ± 4.01	20.67 ± 6.17 *	61.25 ± 44.43 *#	0.004	86.9 ± 89.0	456.6 ± 442.0
cTnI-hs (ng/L)	3.42 ± 1.16	17.83 ± 12.97 *	29.08 ± 18.31 *#	<0.001	435.3 ± 305.6	793.3 ± 487.7
LDH (U/L)	233.8 ± 61.7	348.3 ± 94.5 *	450.5 ± 189.1 *	0.005	56.2 ± 55.2	110.6 ± 116.4
CRP (mg/dL)	0.058 ± 0.05	0.066 ± 0.043	0.697 ± 0.365 *#	<0.001	20.4 ± 30.5	1372 ± 789
IL-6 (pg/mL)	2.07 ± 0.85	50.39 ± 41.58 *	72.08 ± 61.27 *	0.001	2748 ± 2803	3914 ± 4037
IgA (mg/dL)	272.8 ± 163.4	267.5 ± 145.9	279.1 ± 154.2	0.511	1.1 ± 18.1	5.5 ± 16.7
IgM (mg/dL)	139.2 ± 83.2	123.4 ± 68.9	126.7 ± 75.0	0.291	−5.2 ± 19.6	−1.5 ± 23.7
IgG (mg/dL)	1176 ± 302	1193 ± 298	1282 ± 298 *#	0.004	2.2 ± 8.4	11.1 ± 13.0

* Significant difference from PRE, # Significant difference from MID.

## Data Availability

The data presented in this study are available on reasonable request from the corresponding author.

## Abbreviations

The following abbreviations are used in this manuscript:

RBC	Red Blood Cells
HBG	Hemoglobin
HCT	Hematocrit
MCV	Mean Corpuscular Volume
MCH	Mean Corpuscular Hemoglobin
MCHC	Mean Corpuscular Hemoglobin Concentration
WBC	White Blood Cells
NEU	Neutrophils
LYM	Lymphocytes
MONO	Monocytes
EOS	Eosinophils
BAS	Basophils
PLT	Platelets
PCT	Plateletcrit
MPV	Mean Platelet Volume
AST	Aspartate Aminotransferase
ALT	Alanine Aminotransferase
ALP	Alkaline Phosphatase
CK	Creatine Kinase
CK-MB	Creatine Kinase–Myocardial Band
cTnI-hs	High-Sensitivity Cardiac Troponin I
LDH	Lactate Dehydrogenase
CRP	C-Reactive Protein
IL-6	Interleukin-6
IgA	Immunoglobulin A
IgM	Immunoglobulin M
IgG	Immunoglobulin G
